# The Impact of Absorptive Capacity on Innovation: The Mediating Role of Organizational Learning

**DOI:** 10.3390/ijerph19020842

**Published:** 2022-01-12

**Authors:** Rafael Sancho-Zamora, Felipe Hernández-Perlines, Isidro Peña-García, Santiago Gutiérrez-Broncano

**Affiliations:** 1 Department of Business Administration, Faculty of Law and Social Science, University of Castilla-La Mancha, 13003 Ciudad Real, Spain; 2 Department of Business Administration, Faculty of Law and Social Science, University of Castilla-La Mancha, 45071 Toledo, Spain; Felipe.HPerlines@uclm.es; 3 Department of Business Administration, School of Computer Science, University of Castilla-La Mancha, 13003 Ciudad Real, Spain; Isidro.Pena@uclm.es; 4 Department of Business Administration, Faculty of Social Science, University of Castilla-La Mancha, 45600 Talavera de la Reina, Spain; Santiago.Gutierrez@uclm.es

**Keywords:** innovation capacity, absorptive capacity, learning capabilities, small and medium enterprises, knowledge management

## Abstract

Although the relevant literature has already demonstrated the impact that absorptive capacity has on companies’ innovation capacity, we have found few studies that analyze the role of learning capability in this relationship. The main objective of this study was to examine the role of organizational learning in this relationship. For this purpose, a quantitative research approach was used. A total of 306 valid questionnaires were obtained from small and medium-sized Spanish companies in different sectors. The collected data were analyzed using the multivariate Partial Least Square (PLS) quantitative structural equation technique. According to the result, absorption capacity turns into innovation mainly when learning capacity is involved in this process. This study provides empirical evidence of this relationship and fills this gap. It can also help organizations understand and clarify what would be the most appropriate way in to manage knowledge to improve their innovation levels.

## 1. Introduction

In a competitive and changing environment such as that in which we are living at present, permanent and continuous innovation is an increasingly necessary means to ensure the survival of companies [1,2]. Innovation is the main mechanism by which companies produce the new products, processes, and services that allow them to generate competitive advantages [3]. The capacity for innovation allows companies to transform their knowledge into new products or processes so as to clearly improve their market position by responding to the different competitive challenges posed by the current globalized environments [4]. It is innovation that allows companies to produce the new products, processes, and systems that are necessary in order to adapt to markets, technological changes, and new ways of competing [3].

Recent studies show that innovation success depends increasingly on companies’ knowledge management capabilities [5,6,7,8]. This means of improving their knowledge acquisition, learning, and assimilation systems is what will generate greater competitiveness in today’s companies. However, if these companies are to shape their innovation strategies, they not only need to absorb new information from the environment but also use it internally [9].

Previous studies have shown that knowledge sharing and absorptive capacity are the key aspects as regards enhancing innovativeness [10,11,12]. However, if they are to innovate successfully in a highly competitive environment, companies need to combine different types of capabilities [5]. The increase in the number of business opportunities and the increase in competition additionally force companies to combine both internal and external learning processes and thus respond to the demands of the environment through innovation.

Cohen and Levinthal [13,14] introduced the concept of absorptive capacity in order to label all innovation-oriented capabilities. Absorptive capacity focuses particularly on those capabilities that certain firms develop so as to recognize the value of new knowledge and to assimilate and apply it in order to improve their business. It also allows companies to access external knowledge and resources that they cannot generate internally, in addition to developing a more diverse knowledge base, updating their core competencies, and better adapting to external changes. The ability to learn, meanwhile, makes it possible to generate new internal knowledge, to develop the necessary competencies, to understand the process of knowledge development and, together with the knowledge acquired externally, to achieve innovations that are difficult to imitate. In this respect, Kranz, et al. [9] state that when developing innovation strategies, companies need to absorb new information from the environment and use it internally through mechanisms in order to learn, disseminate, and take advantage of the knowledge acquired [15].

Despite the enormous growth in the literature on absorptive capacity, there is still a methodological gap in most studies in terms of a certain amount of ambiguity in the definition and validation of the construct [16]. This situation, therefore, provides abundant opportunities for research in the areas of learning, absorptive capacity, innovation performance, and competitive advantage [11]. It is currently possible to find a growing number of studies that emphasize the extent to which innovation involves the combination of companies’ internal learning capabilities and absorptive capabilities, and our work falls within this field. Despite the existence of literature on the subject, this paper analyzes the role played by organizational learning in the relationship between absorptive capacity and innovation. It thus contributes to the dearth of work that explores in combination how external knowledge acquisition networks, together with internal learning mechanisms, can improve innovation outcomes [11]. This contributes to improving the knowledge management of companies that want to be at the forefront of innovation, as it analyzes the relationship between these variables.

The main objective of this paper is to explicitly address the effect of absorptive capacity on innovation capacity and the role of organizational learning. To this end, we understand innovation capability to be the result of organizational learning carried out by companies [14] and the fact that if these capabilities do not generate new products, services, and processes, neither competitive advantages nor higher performance will be achieved by those companies [17,18]. Innovation involves the generation, acceptance, and implementation of new ideas, processes, products, or services. It is clear that learning orientation is closely related to organizational innovation [19]. Companies must, therefore, have mechanisms in place with which to learn, disseminate, and leverage knowledge in order to drive new innovations [15]. Recent studies such as those of Lane et al. [20] and Song et al. [21] show that external knowledge, and especially that attained from technological advances and market changes, is complementary and should be integrated into organizational learning so as to improve innovation and business performance.

In order to achieve the objectives proposed in this research work, a preliminary study of the theoretical foundations has been carried out, analyzing absorptive capacity as a source of innovation and the role played by organizational learning in this relationship. Subsequently, in order to empirically contrast hypotheses, we have developed a structural equation model based on data collected through a survey of 306 small and medium-sized companies from different manufacturing and service sectors in Castilla-La Mancha. Finally, discussion, limitations, and the most relevant contributions of the study are presented.

## 2. Theoretical Foundations and Hypothesis Statement

### 2.1. Absorptive Capacity as a Source of Innovation Materials and Methods

In many respects, absorptive capacity incorporates the concept of dynamic capabilities introduced by Teece et al. [22], which is a key element as regards obtaining competitive advantages through, among other aspects, greater investment in R&D [12,23]. It was initially defined by Cohen and Levinthal [13] as the ability to attain external knowledge through the processes of the identification, assimilation, and exploitation of knowledge. Subsequently, Zahra and George [24] linked the construct to a set of strategic routines and processes through which firms acquire, assimilate, transform, and apply this knowledge with the aim of creating a competitive advantage [16] based mainly on innovation [10]. This is because new opportunities are explored in the process of adjusting the internal organization to changes in its environment [24]. Furthermore, a company that is committed to the absorption of external knowledge increases its capacity for innovation, since it is less likely to miss opportunities that appear in the market and has a better ability to understand and anticipate its customer needs and discover its competitors’ strengths and weaknesses [25].

This relationship between absorptive capacity and innovation has been studied and empirically confirmed in high-tech companies [26], service companies [27], industrial companies [28], or in multi-sector studies [29]. Jantunen [30] states that most previous work has emphasized the importance of a company possessing external knowledge and its ability to use it, i.e., its absorptive capacity.

Schilling [31] found that absorptive capacity allows companies to expand their knowledge and skill base, improve their ability to assimilate and use future information, and finally, improve the performance of their technological developments. Schmidt and Rammer [32] found that companies with a higher absorptive capacity had a greater possibility of carrying out product, process, organizational, or marketing innovations. When analyzing the different studies that related both variables, Calero Medina and Noyons [33] similarly found that the relationship between absorptive capacity and organizational innovation was significant.

The study carried out by Müller and Bulliga [28] on a sample of 221 German companies showed that companies that acquire, assimilate, transform, and exploit external knowledge are better prepared to develop innovation strategies and apply new business models. In order to acquire external knowledge, it is necessary to recognize and value useful knowledge obtained from the environment. Knowledge assimilation refers to the routines and processes that the company has in place in order to understand, analyze, and interpret knowledge extracted from external sources, while transformation is the act of developing routines with which to combine existing knowledge with newly acquired and assimilated knowledge, and knowledge exploitation refers to applying it commercially in order to achieve business objectives [34].

Using previous theoretical and empirical literature as a basis, we propose the following hypothesis:

**Hypothesis 1 (H1)**. *The greater a company’s absorptive capacity, the greater its innovative capacity*.

### 2.2. The Role of Organizational Learning

Organizational learning has been the subject of study since the 1990s [35] and has been driven mainly by the need to maximize the use of knowledge within organizations [36,37].

Organizational learning can be considered as the way in which organizations learn. This learning refers to any changes in organizational patterns that drive improvements in a company’s performance [38]. Learning capability is specifically a set of intangible resources that the company uses to achieve new forms of competitive advantage [22] and has been considered as a key indicator of organizational effectiveness and innovation potential [39].

For Lemon and Sabota [40], organizational learning is a process with different phases (acquisition, transmission, and use of knowledge) that are closely related to innovation performance. This learning capacity developed by companies has been defined as the capacity to process knowledge, thus incorporating the capacity to create, acquire, transform, and integrate knowledge and to modify the required behavior according to changes in the environment [39].

The concept of organizational learning capability was developed from the literature focused on organizational learning but was quickly linked to the resource- and capability-based approach and to the perspective of organizational capabilities [41]. Learning capability, therefore, requires the integration of other specific capabilities into the company, and its development and implementation over time provides a source of competitive advantage [42]. Recent studies, such as the one conducted by Ober [43], show that there are organizational characteristics that determine success in the adoption of innovations, such as vertical integration, organizational size, openness to new solutions, and/or product diversity.

Absorptive capacity and organizational learning are, therefore, closely linked concepts. More recent definitions of absorptive capacity highlight this relationship when they state that absorptive capacity is a company’s ability to utilize external knowledge through the sequential processes of exploratory, transformative, and exploitative learning [20].

Absorptive capacity consequently depends also on the stability and robustness of organizational learning [44], which enables the organization to leverage the pre-existing knowledge within it, combined with knowledge acquired from external sources, and transform it into new resources and capabilities [14]. Hult et al. [45] point out that, for a company to be innovative, its management must conceive organizational characteristics that incorporate a clear learning orientation.

Despite the existence of this link, the relationship among the aforementioned aspects is currently established with little precision, and researchers have not really been able to understand how these learning processes are carried out by organizations [46]. In this respect, Sun and Anderson [47] analyzed the relationship between absorptive capacity and organizational learning and discovered authors who consider that absorptive capacity is an outcome of organizational learning [29,48]. Others, however, establish absorptive capacity as an antecedent of learning [49,50,51,52,53]. There are also authors who establish a recursive relationship between both concepts [29,48]. Despite this disparity of criteria, this paper establishes that absorptive capacity is an antecedent of organizational learning, because only the works of Szulanski [53] and Reagans et al. [52] empirically tested this relationship [46]. Moreover, we agree with researchers who defend absorptive capacity as an antecedent of learning, because they argue that an organization that has absorptive capacity has a greater capacity to acquire and learn from new sources of knowledge as long as there is a degree of coincidence or overlap with the knowledge it already has.

Studies such as those conducted by Lane et al. [20] and Song et al. [21] show that external knowledge, and especially that attained as a result of technological developments and market changes, is complementary and must be integrated into organizational learning in order to improve innovation and business performance.

In early work related to absorptive capacity, Cohen and Levinthal [13,14] established three key components: recognizing the value of new information, assimilating that information, and applying it for commercial purposes. They also emphasized the value of having external knowledge and not only internally developed knowledge, thus highlighting the important role played by the learning capacity of organizations. It is necessary to be aware that more and more professionals and academics are recognizing the competitive advantages originating from not only internal knowledge but also principally from the absorption of external knowledge [44], which is, in turn, based on learning processes [16,20,54].

Reagans and McEvile [52] stress the importance of having common knowledge, relational embeddedness, and of strengthening ties in order to explain knowledge transfer based on absorptive capacity. To this end, Xerox was a pioneer in developing user interface software technologies, although they were implemented by Apple and Microsoft [55]. These cases reinforce the idea that possessing prior knowledge is not sufficient in order to assimilate and exploit that knowledge for commercial use [47]. Knowledge is assimilated when the organization has an adequate cognitive structure, otherwise that knowledge does not materialize and is wasted. Moreover, the absorption capacity is not static but evolves through the organization’s learning processes [56].

This learning process, which consists of knowledge acquisition, diffusion, and assimilation, is closely related to innovation performance [40,57]. Recent studies have shown that innovation success increasingly depends on the development and integration of knowledge in the innovation process [6,7]. Companies that wish to innovate successfully have to combine different internal learning activities and foster the acquisition and assimilation of knowledge and technology attained from the market [5].

There is growing evidence that learning capability positively influences organizations’ ability to innovate [58,59,60,61,62]. Recent studies, such as those conducted by Migdali [63], Kittikucotiwut [64], Lin et al. [65], Farzaneh et al. [66], Alegre and Chiva [67], and Forés and Camisón [5] highlight the positive effect that learning capability has on innovation.

Given the relationship between absorptive capacity and organizational learning, and of the latter with innovation, we propose the following hypothesis:

**Hypothesis 2 (H2)**. *Organizational Learning mediates the relationship between a company’s absorptive capacity and its innovation*.

**Figure 1:**
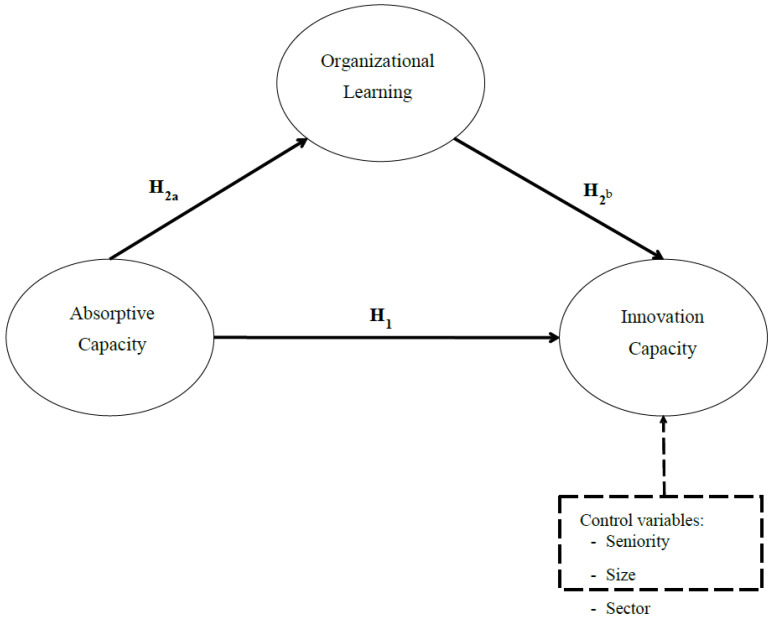
Research Model.

Having carried out the literature review, it is possible to specify the proposed research model (see Figure 1).

## 3. Methodology

### 3.1. Data Collection

The questionnaire was translated from English into Spanish and presented to 5 entrepreneurs to analyze their level of understanding. Once the questions had been adjusted and adapted to the person who was going to answer it, it was sent by email to 800 small and medium-sized randomly selected companies in the Spanish autonomous community of Castilla-La Mancha. The contacts were obtained from the SABI database, and active companies belonging to different sectors of activity in both the industrial and service sectors were selected. A total of 315 questionnaires were obtained, of which 9 were rejected because they were incomplete (see Table 1).

Table 2 shows the sectors and the activity to which the companies that participated in this study belong.

The statistical power of the sample used in this study was 0.998 and was calculated using Cohen’s [69] retrospective test, which can be obtained from the G * Power 3.1.9.2 program [70]. The value obtained makes it possible to state that the sample used in the present study had an adequate statistical power because it was higher than the threshold of 0.80 established by Cohen [69].

### 3.2. Measurement of the Variables

Measurement of absorption capacity. Absorptive capacity was measured by employing the scale of four dimensions suggested by Zahra and George [24] and proposed and validated by Flatten et al. [71]. This second-order composite made it possible to assess the degree to which a company engages in knowledge acquisition (3 items), assimilation (4 items), transformation (4 items), and exploitation (3 items), (i.e., “Management expects employees to have information beyond/outside our industry/sector”).

Measuring innovation capacity. The scale proposed by Prajogo and Soal [72] was used to measure innovation capacity. This second-order composite was applied to two types of innovation: product innovation (measured on the basis of 5 items) and process innovation (4 items or indicators), (i.e., “The company has been the first to introduce a very large number of new products and services”).

Measurement of learning capability. The scales proposed by Goh [73], Jerez et al. [39] and Chiva et al. [36] were used to measure learning capacity, resulting in a first-order composite of 4 items (i.e., “The company has learned or acquired a lot of important new knowledge in the last three years”).

Control variables. In this research, size (number of employees), seniority (number of years since incorporation), and the main sector of activity of the family business were used as control variables, which are recurrently used in studies on family businesses or small and medium enterprises [74].

### 3.3. Results

The hypotheses were tested, and the direct and mediating effects were analyzed by employing the Partial Least Square (PLS) multivariate quantitative structural equation technique. The choice of this data analysis method is justified for the following reasons: (1) it is an appropriate method in the early stages of a new theory [75,76,77], (2) its predictive nature makes it possible to address the research questions in hand [78,79], (3) it allows the observation of different causal relationships [80,81], and (4) it is a suitable method when the sample size is small [82,83].

The software used for data analysis via SEM-PLS was SmartPLS v.3.3.3 (SmartPLS GmbH, Pinneberg, Germany) [76]. SEM-PLS is a multivariate analysis technique whose purpose is to test structural models, its main objective is cause–effect analysis [84]. In our case, the proposed model tries to test how absorptive capacity is an antecedent of innovativeness, i.e., that absorptive capacity positively influences innovativeness. In addition, learning capability is included in the model as a mediating variable. This implies that the influence of absorptive capacity on innovativeness increases through the mediation of learning capacity.

Among the various SEM techniques, in this paper we have opted for the PLS following the recommendations of Hair et al. [85]. For these authors, PLS is a more flexible technique that does not require the data to follow a normal distribution. Moreover, it is a non-parametric method whose use is recommended when the measurement scale is the ordinal Likert scale, as in our case. On the other hand, the aim of this work is to test how absorptive capacity influences innovation capacity, either through a direct relationship or through a relationship mediated by learning capacity. In other words, we try to analyze the explained variance of innovativeness. The PLS method allows us to maximize the coefficient of determination (R2).

We first verified that the variables were reliable and had adequate levels of convergent and discriminant validity. This took place after operationalizing absorptive capacity as a second-order type A composite and both learning capacity and innovativeness as first-order type A composites. We used a traditional PLS to model these type A composites, following the method described by Henseler et al. [86].

The following indicators with which to perform the evaluation of the measurement model were proposed by Barclay et al. [87], Roldan and Sanchez-Franco [88], and Hair et al. [85]:

- Composite reliability—Fornell and Larcker [89] recommend values above 0.7 for composite reliability. The observed values were, according to the Hair et al. [78] criteria, adequate because they were between 0.7 and 0.9. Moreover, there were no redundancy issues because no value was higher than 0.95 [90,91]. All the indicators in the model had acceptable composite reliability values (see Table 3).
- Cronbach’s alpha—Forner and Larcker [89] recommend Cronbach’s alpha values greater than 0.7. As Table 2 shows, Cronbach’s alpha was higher than this value for all the variables.
- Rho a—According to Dijkstra and Henseler [92], Rho a must be greater than 0.7 and must lie between the composite reliability and Cronbach’s Alpha values [78]. This condition was met for our data (see Table 3).
- Average variance extracted (AVE) can be used to assess the convergent validity of each composite. Forner and Larcker [89] recommend a value higher than 0.5 for the AVE. This condition was valid for our data (see Table 3).
- Discriminant validity can be analyzed by checking that the correlation between each pair of constructs is not greater than the square root value of the AVE for each construct and using the heterotraitmonotrait ratio (HTMT). For discriminant validity to hold, HTMT values must be less than 0.85 [86]. These conditions held for the data, thus confirming discriminant validity (see Table 3).

The three variables analyzed (absorptive capacity, learning capacity, and innovative capacity) are highly correlated as they are dynamic capabilities, which could lead to the appearance of a common method bias. In our work, in order to avoid this bias, we have carried out a confirmatory factor analysis in order to assess whether the variance in the data could be attributed to a single factor. The results show values close to 0, specifically 0.0001, so we can affirm that there is no common bias due to the method.

In order to complete the verification of discriminant validity, we also calculated the HTMT inference from the bootstrapping option (5000 subsamples). When the resulting interval contain values lower than 1, there is discriminant validity, and our data fulfilled this requirement (see Table 4).

With the previous analyses, we have been able to verify that the measurement model has the necessary convergent and discriminant validity. Once this was verified, we carried out the study of the relationships between the variables incorporated in the structural model, which includes both the direct effect and the mediating effect. To do so, the steps proposed by Hair et al. [93] were followed to apply the approach of Preacher and Hayes [94].

**Figure 2:**
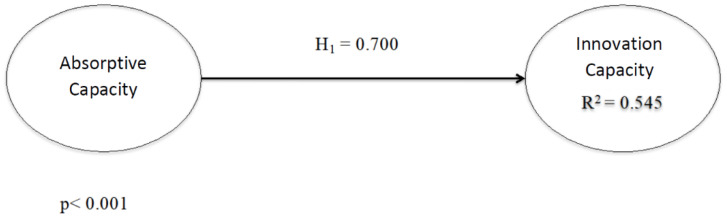
Direct model.

To analyze the direct effect between absorptive capacity and innovativeness, the value of the path coefficient and its significance were tested. The bootstrapping procedure of 5000 subsamples was followed, which is the usual procedure in these cases. The effect was positive and significant (β = 0.700; *p* < 0.001) (see Figure 2 and Table 5). Moreover, this result was reinforced by applying the percentile method in bootstrap resampling at the 95% confidence level (see Table 5). Finally, absorptive capacity was able to explain 54.5% of the variance of innovativeness.

**Figure 3:**
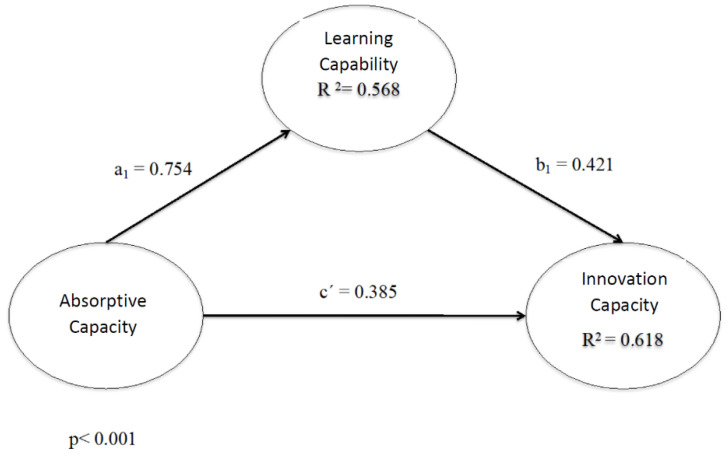
Mediation model.

The second step was that of including the effect of the mediating variable (learning ability). It will be noted that absorptive capacity has a positive and significant influence on learning capacity (a1 = H2a: β = 0.754; *p* < 0.001), with the former being able to explain 56.8% of the variance of the latter (see Figure 3 and Table 4). Furthermore, learning ability is a positive and significant antecedent of innovativeness (a2 = H2b: β = 0.754; *p* < 0.001), and is able to explain 61.8% of its variance (see Figure 3 and Table 5).

There is, therefore, a mediation of learning ability in the relationship between absorptive capacity and innovativeness, but this effect is not total, as the direct effect is not suppressed [95,96] because absorptive capacity remains a positive and significant antecedent, even if the mediation of learning ability occurs (β = 0.385; *p* < 0.001) (see Table 5).

Once we have analyzed the influence that the control variables may have, none of them is considered relevant (the path coefficients are less than 0.2), and they are not significant (their value is lower than the recommended value (*p* < 0.001).

When comparing the two models and considering the quality parameters, we can state that the mediation model has a better fit value than the direct model, as it obtains a better SRMR (normalized root mean square residual) value. While the direct model obtains an SRMR of 0.071, the mediation model obtains an SRMR of 0.067. Both models are below the threshold set by Henseler et al. [86], thus validating the mediating role of learning capability in the relationship between absorptive capacity and innovation.

## 4. Conclusions

Based on a review of the theoretical and empirical literature, this paper has attempted to advance the study and understanding of organizational absorptive capacity. It shows that it is a construct that contributes to a large extent to understanding the functioning and behavior of the organization in an increasingly dynamic, complex, and competitive environment, especially when it comes to learning, innovation, and knowledge management processes. Different previous studies, as well as those provided in this study, show that companies with a greater absorption capacity make better use of the information they receive from outside and also improve their capacity for innovation. Mainly in highly changing environments, this circumstance is fundamental for the improvement of their processes and products with the aim of improving their competitive position with respect to the rest of their competitors.

Although the relevant literature has already demonstrated the impact that absorptive capacity has on companies’ innovation capacity, we found few studies that analyze the role played by learning capacity in this relationship. We, therefore, consider that one of the main contributions of this study was to provide empirical evidence of this relationship and to fill this gap. It may also help organizations understand and clarify what would be the most appropriate way in which to manage knowledge in order to improve their innovation levels.

The concept of absorptive capacity makes it possible to understand the internal and external dynamics of organizations and their learning [97]. However, despite the fact that since [14]’s contribution to the concept, several contributions have been made in an attempt to strengthen its explanatory and descriptive character [24], the conceptualizations in this analytical field have generally lacked a more thorough reflection on the innovation-knowledge relationship and on the forms of learning that interact in this relationship. In order to boost the potential of the concept of absorptive capacity, we consider it necessary to nurture it with concepts developed in other fields of research, such as innovation itself and learning.

The literature on knowledge management produced to date is not sufficiently clear as regards the internal process that occurs in order to transform this knowledge into innovation, but our study helps better understand what occurs in those organizations that innovate most. As we can see in Table 3, the mean of the three variables considered in the model is high, above 4, especially the learning capability. These results together with the high correlation between the variables considered reveal that high values of absorptive capacity are associated with high values of innovative capacity and that the values of the latter are higher when learning capacity is incorporated. With all of the above, the positive mediating effect of learning capacity on the influence of absorptive capacity on innovation capacity is observed, as already suggested by the results of the PLS.

There are numerous studies linking ACAP and OL. However, the distinction or relationship between the two concepts has not been carefully articulated. We, therefore, consider that one of the main contributions of this study is to provide empirical evidence of this relationship and to fill this gap. Specifically, this study shows that the ACAP has a positive influence on OL. This imply that an organization’s ACAP facilitates its ability to acquire and learn from new sources of knowledge as long as a degree of commonality or knowledge overlap exists. These results are consistent with the contribution of Lichtenthaler [54] who identifies technological and market knowledge as critical components in the organizational learning process.

Another contribution of this paper to the existing literature is the discovery that absorption capacity becomes innovation mainly when learning capacity is involved in this process. The model offers an integrated view of the overall innovation process, which is understood as a complex process that requires the use of external and internal information. We have specifically found that the relationship between absorptive capacity and innovative capacity is partially mediated by companies’ learning capacity. Keeping in mind that the concept of absorptive capacity is a phenomenon that has become a point of analysis by which to approach the study of innovation processes, Vega-Jurado et al. [98] mention in this regard that external knowledge is not applicable if the company does not develop new competencies that allow it to increase its capacity to assimilate it.

### 4.1. Practical Implications

This work has also revealed some implications for firms. It is clear from this research that the firm needs to improve its capacity to acquire, assimilate, and transform external knowledge if it wants to improve its capacity to innovate. To do so, it must adapt its human capital as a key element to increase innovation through knowledge assimilation. Moreover, when organizations increase their learning capacity, the impact that the acquisition of external knowledge has on business innovation increases, so it is necessary for companies to have flexible internal structures and continuous learning systems that allow them to integrate this new knowledge into the existing business structure, thus creating up-to-date and available knowledge that can be used by the entire organization.

Finally, once this new knowledge has been acquired by the firm’s absorptive capacity and integrated into the organization’s learning system, it is ready to enhance the firm’s capacity for innovation to a much greater extent, thus generating improvements in the firm’s internal processes and in its capacity to design, manufacture and market new products and services, thus improving its competitiveness and adaptation to its environment.

### 4.2. Limitations and Future Research

Although this paper provides empirical evidence to corroborate the selected working hypotheses, we find some limitations. Firstly, it only and exclusively analyzes the impact that absorptive capacity has on learning capacity and innovation. Innovation, however, involves more capabilities that could be analyzed in the future. Secondly, this study has a regional character, which could be extended in the future. Given the global nature of the economy, it is imperative to test the applicability of the learning and innovation constructs in broader and more diverse contexts. Finally, the use of a single informant has been criticized by several authors, although in our study the informant was the company director, who is therefore a highly qualified person to provide the information.

Future research could use PLSpredict, although researchers using PLS-SEM emphasize the predictive nature of their analyses. The evaluation of the model is based exclusively on metrics designed to assess the explanatory power of the trajectory model, and in this sense, PLSpredict generates predictions at the item or construct level [99]. Finally, as in all cross-sectional research, hypothesis testing was conducted at one point in time although these capabilities have undoubtedly been developed over time by the companies. This shows that even small and medium-sized enterprises are capable of acquiring dynamic capabilities that are useful and necessary for their survival.

Although it is likely that the conditions under which the data were collected will not change substantially, there is no guarantee that this will be the case, and therefore researchers must carefully interpret causality between constructs.

## Figures and Tables

**Table 1:** Research technical data.

Population/Sample Size	15.853 Companies/800 Randomly Selected Companies
Unit of analysis	Company
Scope	Castilla-La Mancha (Spain)
Valid responses/Response rate	306/38.25%
Confidence level	95%
Error rate	5.55%
Informant	CEO
Data	October–December 2019

Reprinted from ref. [68].

**Table 2:** Sector and activity of the analyzed companies.

Sectors (CNAE)	Code	Activity	Number	Percentage
62, 69, 70, 71, 73	1	Specialized consulting services	75	24.50%
41, 43	3	Construction	65	21.24%
55, 56, 46, 47, 68	2	Retail and accommodation services	96	31.37%
10, 11, 14, 18, 21, 23, 25, 26, 27, 28, 31	4	Manufacturing	70	22.87%
			306	

Reprinted from ref. [68].

**Table 3:** Correlation matrix, composite reliability, convergent, and discriminant validity, Heterotrait-monotrait ratio (HTMT) and descriptive statistics.

Construct	AVE	Composite Reliability	ACAP	LC	IC
1. Absorptive capacity (ACAP)	0.834	0.953	0.913 *		
2. Learning capability (LC)	0.661	0.854	0.754	0.813 *	
3. Innovation capacity (IC)	0.766	0.921	0.725	0.733	0.875 *
Heterotrait-monotrait rate (HTMT)					
1. Absorptive capacity					
2. Learning capability			0.842		
3. Innovation capacity			0.765	0.822	
Cronbach’s Alpha			0.934	0.782	0.905
Rho A			0.936	0.830	0.915
Mean			4.17	4.37	4.22
SD			1.16	1.08	0.92

(*) The values of the diagonal have been obtained from the square root of the AVE of each compound.

**Table 4:** HTMT inference.

	Original Sample (O)	Sample Mean (M)	5.0%	95.0%	Sample Mean (M)	Bias	5.0%	95.0%
ACAP -> IC	0.385	0.394	0.282	0.508	0.336	0.009	0.265	0.489
ACAP -> LC	0.754	0.752	0.282	0.693	0.802	0.002	0.693	0.802
LC -> IC	0.421	0.421	0.082	0.324	0.523	0.000	0.324	0.523

**Table 5:** Causal relationships: total, direct, and indirect effects.

	Coefficient Path (β)	*t*-Value (Bootstrap)	Confidence Intervals 95%		Support
			5%	95%	
SRMR: 0.071					
Total effect (ACAP-IC) (*c)*	0.700 ***	13.547	0.607	0.777	Yes
SRMR: 0.067					
ACAP → IC = *c’* (direct effect)	0.385	5.660	0.310	0.536	Yes
H_1_ = ACAP → LC → CI = *a*_1_*b*_1_ (total indirect effect)	0.317		0.264	0.487	Yes
H_2_ = ACAP → LC = *a*_1_	0.754 ***	22.401	0.693	0.803	Yes
H_3_ = LC→ IC = *b*_1_	0.421 ***	6.984	0.323	0.519	Yes

*** *p* < 0,001 *t*-Student distribution (one tail, 4999 degrees of freedom).

## Data Availability

Data sharing not applicable.
